# The crucial question: does adjuvant radiotherapy confer a survival benefit in pN1 oral squamous cell carcinoma (OSCC)? - A retrospective analysis

**DOI:** 10.1007/s10006-026-01654-1

**Published:** 2026-10-24

**Authors:** Maximilian Richter, Aleksandra Tworek, Steffen Koerdt, Max Heiland, Leonard Knoedler, Daniel Zips, Marcus Beck, Friedrich Mrosk

**Affiliations:** 1 Department of Oral and Maxillofacial Surgery, Campus Virchow-Klinikum, Charité - Universitätsmedizin Berlin, corporate member of Freie Universität Berlin and Humboldt-Universität zu Berlin, Augustenburger Platz 1, 13353 Berlin, Germany; 2 Department of Radiation Oncology and Radiotherapy, Charité - Universitätsmedizin Berlin, corporate member of Freie Universität Berlin and Humboldt-Universität zu Berlin, Augustenburger Platz 1, 13353 Berlin, Germany

**Keywords:** Oral squamous cell carcinoma, Prognostic marker, pN1, Adjuvant therapy, Prognosis, Surgical treatment, OSCC

## Abstract

**Objective:**

Despite continuous research on the role of adjuvant therapy in the pN1 setting in Oral Squamous Cell Carcinoma (OSCC), clear recommendations for or against its use remain lacking. The objective of this study is to further strengthen the existing evidence base and support informed clinical decision-making.

**Materials and methods:**

This retrospective analysis included all patients with primary pT1–2 pN1 OSCC without extracapsular spread who underwent surgical treatment at the Department of Oral and Maxillofacial Surgery, Charité – Universitätsmedizin Berlin. Survival analyses were performed using Kaplan–Meier methods and Cox regression models for the overall cohort as well as after propensity score matching (PSM).

**Results:**

A total of 58 patients (male: 34 (58.6%), female: 24 (41.4%)) with a mean age of 63.6 years were included. In the overall cohort, there were no significant differences in overall survival (OS) or recurrence free survival (RFS) between patients treated with surgery alone and those who received adjuvant therapy (*p* = 0.628; *p* = 0.244). In contrast, following PSM, adjuvant therapy was associated with a reduced risk of recurrence, as reflected by improved RFS (HR 0.21 (CI: 0.02–0.85, *p* = 0.029)).

**Conclusion:**

In summary, our findings suggest an association between adjuvant therapy and improved RFS in patients with T1–2 pN1 OSCC, indicating a potential oncological benefit that should be considered in the context of individual clinical characteristics and balanced against potential treatment-related adverse effects.

## Introduction

With an incidence of 389,485 cases and an annual mortality of 188,230 patients, oral squamous cell carcinoma (OSCC) remains a major challenge for the global healthcare system [1]. Despite considerable advances in therapeutic strategies over recent decades, overall survival has plateaued at approximately 56%, underscoring the urgent need for reliable and clinically relevant prognostic markers [2, 3].

Surgical resection remains the widely accepted gold standard for primary treatment of OSCC [4, 5]. However, the role of definitive radiochemotherapy as an alternative primary modality has increasingly been explored, although current evidence does not support its equivalence to surgery [4, 6, 7]. In contrast, there is strong and consistent evidence supporting the use of adjuvant radiotherapy or radiochemotherapy in the postoperative setting [8]. The benefit of adjuvant treatment is well established in the presence of adverse pathological features. According to the National Comprehensive Cancer Network (NCCN) and German clinical guidelines, these include extranodal extension, positive or close surgical margins, pT3 or pT4 primary tumors, pN2 or pN3 nodal disease, involvement of lymph nodes in levels IV or V, perineural invasion, vascular invasion, and lymphatic invasion [5, 9].

While clear recommendations exist for adjuvant therapy in patients with these high-risk features, the evidence regarding pN1 disease remains inconclusive [5]. According to the current classification, pN1 status is defined as a single lymph node metastasis measuring up to 3 cm without evidence of extracapsular extension and is observed in approximately 17.5% of OSCC cases [10, 11]. Despite its relatively frequent occurrence, there is currently no consensus on the indication for postoperative radiotherapy or radiochemotherapy in this subgroup, and further studies are warranted to better define its clinical relevance [5, 9]. While some studies have demonstrated a prognostic benefit of adjuvant therapy in pN1 patients [12–14], others have reported no significant advantage [15], highlighting the ongoing controversy.

A large prospective, multicenter cohort study involving 209 patients from 27 centers demonstrated an improvement in progression-free survival, whereas no significant effect on overall survival was observed [16]. The authors concluded that the potential benefits of adjuvant therapy must be carefully balanced against the risk of radiation-induced toxicity [16].

Despite the strengths of this large prospective study, several limitations should be acknowledged, including tumor classification based on the 7th edition staging system and the inclusion of both OSCC and oropharyngeal carcinomas (OPSCC) [16]. Given the higher radiosensitivity of OPSCC - particularly in the presence of p16 positivity - the direct comparison of these two entities appears questionable [17, 18].

Given the limited and conflicting data on the prognostic impact of pN1 status in surgically treated OSCC patients, the question arises whether its integration into routine clinical decision-making is justified. Therefore, the aim of the present study was to evaluate the prognostic significance of pN1 status in a representative cohort of surgically treated pT1 and pT2 OSCC patients.

## Materials and methods

### Ethics statement

The Ethics Committee of the Faculty of Medicine Charité - Universitätsmedizin Berlin approved this study.

### Patient cohort

This study included all patients diagnosed with pT1, pT2 pN1 OSCC without extracapsular spread (ECS) who underwent surgical treatment at the Department of Oral and Maxillofacial Surgery, Charité – Universitätsmedizin Berlin, between 2009 and 2020. Tumor staging was performed according to the 7th and 8th edition of the American Joint Committee on Cancer (AJCC) staging manual [19, 20]. Clinical and histopathological data, including TNM classification and tumor grade, were collected retrospectively. Additional patient information—such as age, sex, tumor site, treatment modality, survival time, and recurrence status—was obtained from electronic medical records.

Overall survival (OS) was defined as the time from the date of initial diagnosis to death from any cause. Patients without an event were censored at the date of last follow-up or at the end of the observation period (May 10th, 2023), whichever occurred first. In cases where the exact date of initial diagnosis was unavailable (e.g., for externally diagnosed patients), the date of surgical treatment was used as a surrogate. Recurrence-free survival (RFS) was defined as the time from completion of primary therapy to the occurrence of recurrence.

### Statistical analysis

Data collection was performed using Microsoft Excel (Microsoft Corporation, Redmond, WA, USA), while all statistical analyses were carried out with IBM SPSS Statistics (Version 29.0.2.0) and R (Version 4.0.3) [21]. Categorical variables are reported as absolute frequencies and percentages, whereas continuous variables are presented as means with corresponding standard deviations (SD). Comparisons between groups for categorical and ordinal variables were conducted using either the chi-square test or Fisher’s exact test, depending on the data distribution and sample size.

OS and RFS were evaluated using the Kaplan–Meier method, with group differences assessed by the log-rank test. To identify potential prognostic factors for OS and RFS, univariate and multivariate Cox proportional hazards regression models were applied. All p-values were interpreted as exploratory and were not adjusted for multiple testing; a significance level of α = 0.05 was applied. Hazard ratios (HRs) and mean survival times are reported along with their respective 95% confidence intervals (CI).

Propensity score matching was performed using the Propensity Score Matching extension for IBM SPSS Statistics (version 2.0.1), based on the Python programming language. Patients were matched in a 1:1 ratio using propensity score matching, with exact matching (0 deviation) for tumor grade, sex, and pT stage. Balance between groups was subsequently assessed using the standardized mean difference (SMD). In accordance with Cohen’s criteria, an SMD < 0.2 was considered indicative of adequate balance [22].

## Results

### Clinicopathological features

A total of 58 patients with surgically treated pT1-2, pN1 OSCC without ECS were included in this study, comprising 34 males (58.6%) and 24 females (41.4%). The mean age of patients was 63.6 years (SD ± 10.9 years), with a range from 45 to 91 years. As previously described, all patients underwent neck dissection as part of their surgical treatment (unilateral: 63.8%; bilateral: 36.2%), wherein 75.9% of patients received a selective neck dissection, while 24.1% underwent a modified radical neck dissection.

In our cohort, 42 (72.4%) patients received surgery alone, whereas 16 (27.6%) patients underwent additional adjuvant therapy. Correlation analyses revealed no significant differences regarding clinicopathological characteristics between the two groups (Table 1).

**Table 1:** Clinicopathological characteristics of OSCC patients in relation to the treatment modality

		Treatment modality		
		Surgery	PORT	
	total	42 (72.4%)	16 (27.6%)	p-value
Age at surgery		65.0 years	59.9 years	0.116
Sex				
Female	24	18 (42.9%)	6 (37.5%)	0.773
Male	34	24 (57.1%)	10 (62.5%)	
Neck-Dissection Level				
<3 level	26	21 (50.0%)	5 (31.3%)	0.280
4 level	18	13 (31.0%)	5 (31.3%)	
>5 level	14	8 (19.0%)	6 (37.5%)	
History of Smoking				
Yes	34	22 (52.4%)	12 (75.0%)	0.145
No	24	20 (47.6%)	4 (25.0%)	
History of Alcohol				
Yes	21	13 (31.0%)	8 (50.0%)	0.360
No	37	29 (69.0%)	8 (50.0%)	
Grading				
G1	1	0	1 (6.3%)	0.186
G2	41	32 (76.2%)	9 (56.3%)	
G3	15	9 (21.4%)	6 (37.5%)	
G4	1	1 (2.4%)	0	
pT stage				
pT1	26	22 (52.4%)	4 (25.0%)	0.080
pT2	32	20 (47.6%)	12 (75.0%)	

### Survival analysis according to treatment modality

In the overall cohort, mean OS was 131.3 months (CI: 117.7–145.0 months), while RFS demonstrated a mean of 104.2 months (CI: 86.4-121.7 months). The 5-year overall survival rate was 93%.

**Fig. 1:**
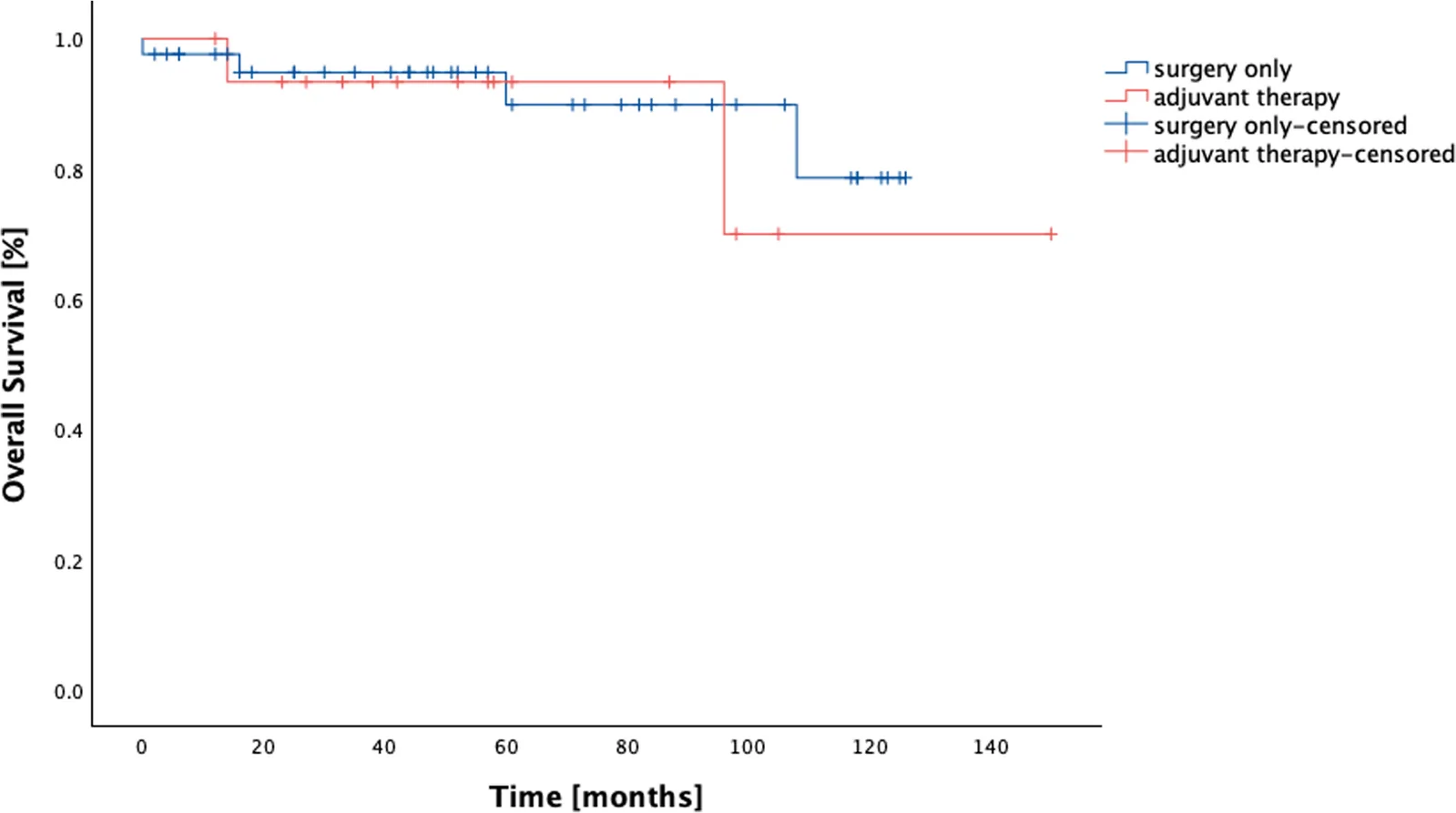
Kaplan–Meier curves of OS stratified by treatment modality, showing no statistically significant difference (*p* = 0.628)

In the subgroup analysis, no significant differences in OS were observed. The mean OS was 114.6 months (CI: 104.1-125.2 months) in the surgery-alone group, compared to 128.3 months (CI: 101.9-154.7 months) in the adjuvantly treated group (*p* = 0.628) (Fig. 1). The 5-year survival rates were 94% and 91%, respectively.

**Fig. 2:**
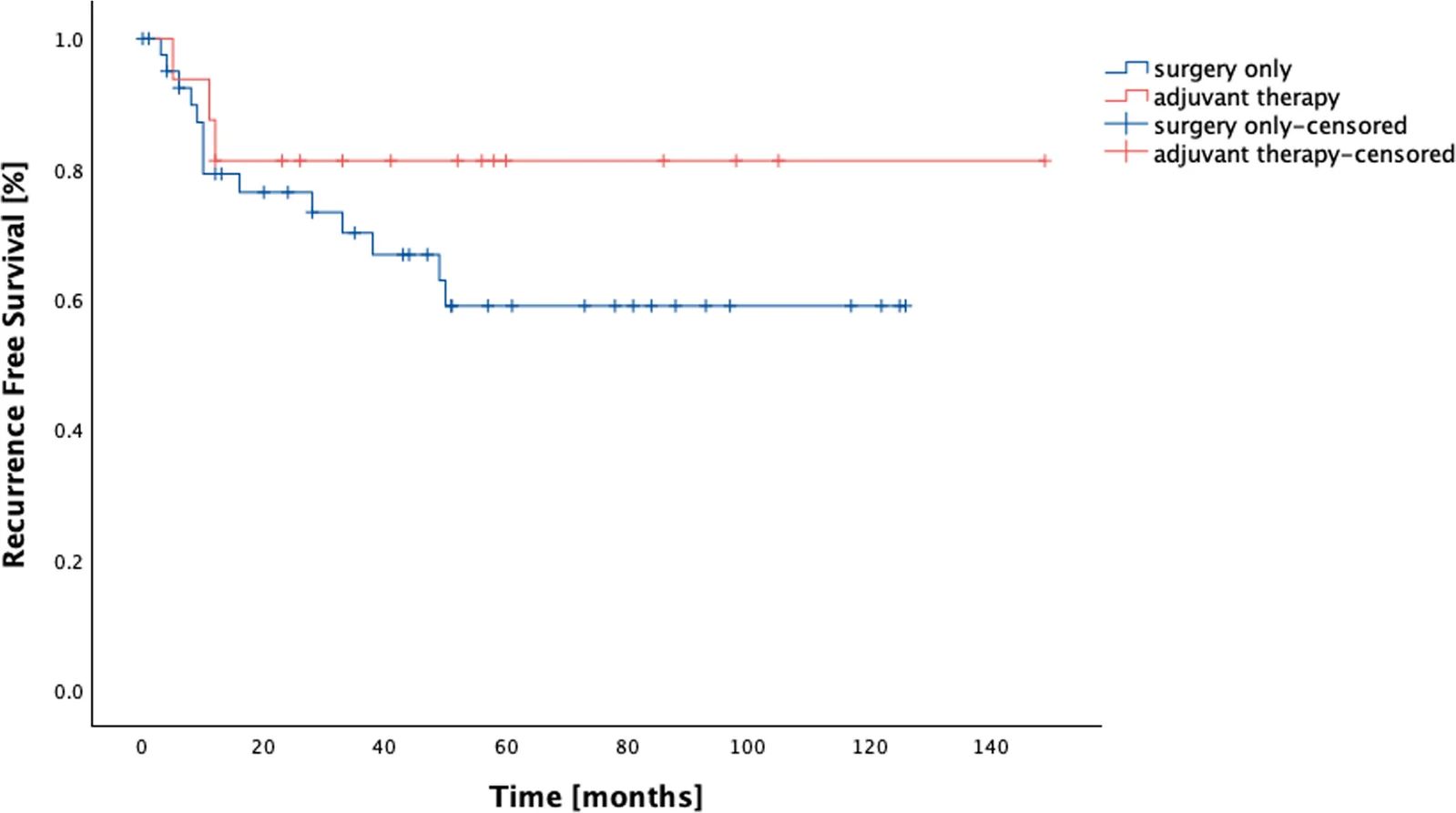
Kaplan–Meier curves of RFS stratified by treatment modality, showing no statistically significant difference (*p* = 0.244)

With regard to RFS, a clear trend in favor of patients receiving adjuvant therapy was observed. These patients demonstrated a mean RFS of 122.8 months (CI: 96.1-149.5 months), compared to 83.4 months (CI: 65.8-100.9 months) in those treated with surgery alone (Fig. 2). However, this difference did not reach statistical significance (*p* = 0.244).

### Survival analysis according to treatment modality after propensity score matching

As outlined in the Materials and Methods, further analyses were conducted after PSM. Of the 58 patients included in the initial cohort, 30 patients were included in the propensity score-matched analyses, comprising 14 patients who underwent surgery alone and 16 patients who received postoperative radiotherapy. Accordingly, 28 patients were not included in the matched analyses due to the absence of an appropriate match. Post-matching assessment revealed no evidence of imbalance between the groups (Table 2).

**Table 2:** Clinicopathological characteristics of OSCC patients in relation to the treatment modality after Propensity Score Matching

		Treatment modality		
		Surgery	PORT	
	total	14 (46.7%)	16 (53.3%)	SMD
Sex				
Female	11	5 (35.7%)	6 (37.5%)	0.02
Male	19	9 (64.3%)	10 (62.5%)	
Grading				
G1 + G2	19	9 (64.3%)	10 (62.5%)	0.02
G3 + G4	11	5 (35.7%)	6 (37.5%)	
pT stage				
pT1	8	4 (28.6%)	4 (25.0%)	0.09
pT2	22	10 (71.4%)	12 (75.0%)	

**Fig. 3:**
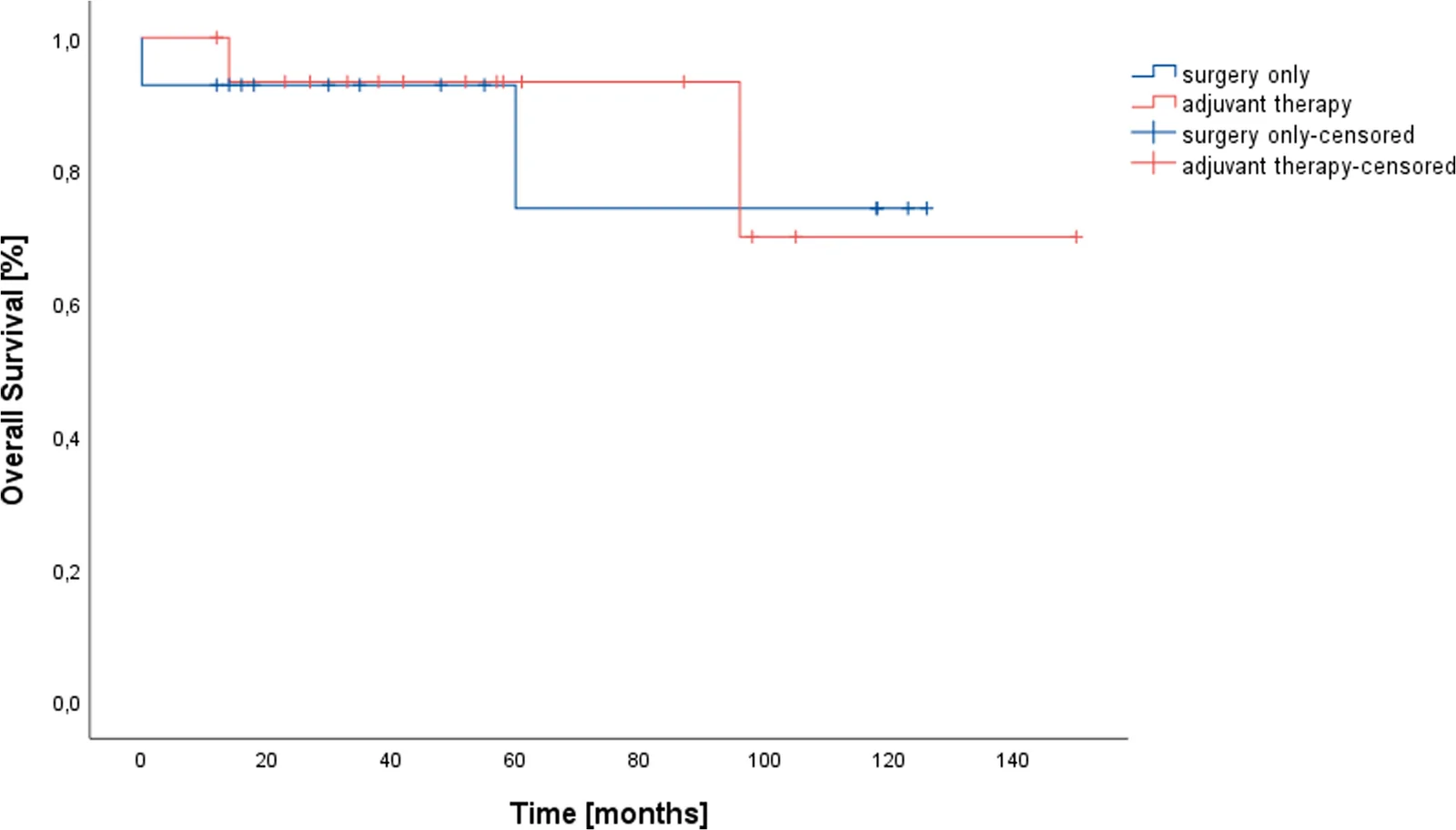
Kaplan–Meier curves of OS stratified by treatment modality after PSM, showing no statistically significant difference (*p* = 0.893)

In terms of OS in the matched cohorts, a trend toward improved survival was observed in patients receiving adjuvant therapy (128.3 months, CI: 101.9-154.7 months), whereas patients without adjuvant treatment demonstrated a mean survival of 104.7 months (CI: 78.4-131.1 months) (Fig. 3). However, this difference did not reach statistical significance (*p* = 0.893).

**Fig. 4:**
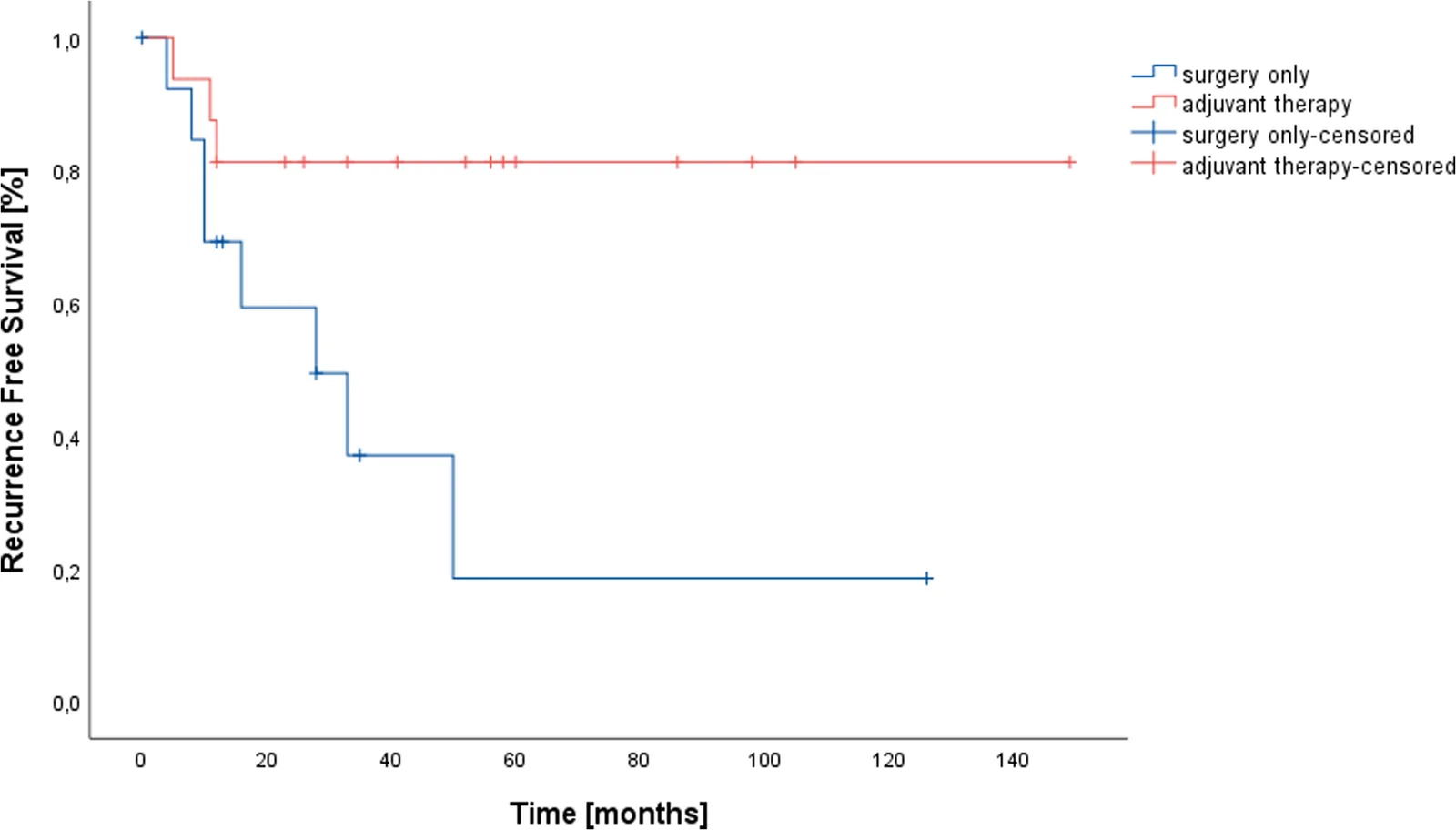
Kaplan–Meier analysis of RFS after PSM demonstrating superior outcomes in the adjuvantly treated cohort (*p* = 0.015)

Regarding RFS, a statistically significant advantage was observed for the adjuvantly treated group (122.8 months, CI: 96.1-149.5 months) compared to patients who underwent surgery alone in univariate analysis (43.5 months, CI: 14.7–72.3 months, *p* = 0.015) (Fig. 4). A multivariable Cox regression model was fitted using forced entry of all prespecified covariates, irrespective of their statistical significance in univariable analysis. Adjuvant therapy was significantly associated with RFS (Table 3). Specifically, patients underwent adjuvant radiotherapy demonstrated a reduced risk with a HR of 0.21 (CI: 0.02–0.85, *p* = 0.029). In contrast, none of the other variables demonstrated a statistically significant impact on RFS.

**Table 3:** Univariate and multivariate cox regression analysis for RFS after PSM

	Univariate analysis			Multivariate analysis		
	HR	95%-CI	*p*-value	HR	95%-CI	*p*-value
Sex						
male	1			1		
female	1.04	0.30–3.56	0.949	1.38	0.34–5.60	0.650
History of Smoking						
Yes	1					
No	0.45	0.14–1.49	0.192			
History of Alcohol						
Yes	1					
No	0.28	0.06–1.29	0.102			
Grading						
G1 + G2	1			1		
G3 + G4	0.70	0.18–2.63	0.592	0.65	0.14–3.08	0.586
pT Stage						
T1	1			1		
T2	0.69	0.20–2.36	0.553	0.67	0.17–2.64	0.565
Adjuvant Radiotherapy						
Yes	0.22	0.06–0.84	0.027	0.21	0.02–0.85	0.029
No	1			1		

## Discussion

The aim of this study was to evaluate the prognostic significance of pathological N1 status in a cohort consisting exclusively of patients with OSCC that were treated surgically.

With regard to OS, no significant impact of adjuvant therapy was observed, neither in the entire cohort nor in the subgroup analyses. These findings are consistent with previously published literature. For instance, a German multicenter study by Kämmerer et al. likewise demonstrated no significant effect of postoperative adjuvant therapy on overall survival [16] (Table 4). Similar conclusions were reported in a multicenter study from China investigating the impact of adjuvant radiotherapy on survival in pN1 OSCC patients without adverse pathological features [15]. The authors concluded that adjuvant therapy does not influence prognosis in T1–2 N1 M0 tumors without adverse pathological features, whereas it may be beneficial in locally advanced disease [15]. In contrast, some studies have reported improved outcomes with adjuvant therapy in the pN1 setting. For instance, a population-based study by Doll et al. demonstrated improved OS in patients with T1–2 OSCC and p16 negative OPSCC in multivariable analysis [13]. Similarly, a retrospective analysis by Struckmeier et al. demonstrated a clear trend toward improved OS in the adjuvantly treated group, although this did not reach statistical significance [12].

With regard to RFS, our cohort demonstrated a trend toward improved outcomes with adjuvant radiotherapy in the overall population. After PSM, this difference reached statistical significance, with superior outcomes observed in the cohort receiving adjuvant therapy. Similar findings were reported by Kämmerer et al., who described improved locoregional control with adjuvant treatment [16]. In contrast, the aforementioned study by Li et al. found no significant prognostic impact on DFS [15].

However, a recently published study with a design comparable to our cohort likewise demonstrated a significantly improved RFS in patients with T1–2 pN1 OSCC [12]. These findings are further supported by a large database study showing comparable oncological outcomes between pN1 and pN0 tumors, but only when pN1 patients received postoperative radiotherapy [23]. Nevertheless, the inherent limitations of large-scale database analyses must be considered, which preclude definitive conclusions.

When interpreting these findings in the context of existing literature, it is essential to consider the specific tumor entities included in the respective studies. While Li et al. exclusively analyzed OSCC patients, other studies included both OSCC and OPSCC [13, 16]. The higher radiosensitivity of OPSCC may, at least in part, account for these divergent results.

**Table 4:** Selection of studies published since 2021 investigating the effect of adjuvant therapy in pN1 setting

	Tumor	Study design	Sample size	OS	DFS/RFS
Kämmerer 2023 [16]	OSCC/OPSCCpT1 + pT2	PMS	209	HR 0.98 (0.55–1.73)	HR 0.41 (0.19–0.89)
Li 2025 [15]	OSCC pT1 + pT2	MCS	231	73.1% vs.75.0%	59.5% vs. 58.3%
Struckmeier 2026 [12]	OSCC pT1-pT4	RA	98	HR 0.50 (0.24–1.06)	HR 0.28 (0.11–0.74)
Xiang 2021 [23]	OSCC pT1 + pT2	RS	530	HR 0.66 (0.46–0.97)	-
Doll 2025 [13]	OSCC/OPSCC pT1 + pT2	RS	526	HR 0.49 (0.32–0.76)	-
Fan 2022 [24]	OSCC pT1 + pT2	RS	469	HR 0.56 (0.34–0.91)	-
Tsai 2021 [14]	OSCC pT1 + pT2	RS	701	HR 0.72 (0.54–0.97)	HR 0.64 (0.48–0.84)
Alsharif 2022 [25]	OSCC pT1 + pT2	PCS	51	HR 0.22 (0.05–1.03)	HR 0.06 (0.11–0.31)

Footnote Table 4: *PMS *Prospective multicenter study, *MCS *Multicenter cohort study, *RA *Retrospective analysis, *RS *Registry-based study, *PCS *Prospective cohort study

In the era of a multidimensional approach to oncologic diseases, not only survival outcomes but also patient-reported factors such as quality of life and treatment-related complications have gained increasing importance. Both radiotherapy and chemotherapy are associated with substantial adverse effects that may have a lasting impact on patients’ quality of life [26, 27]. However, these aspects are often not adequately addressed in most studies.

In contrast, the study by Kämmerer et al. also evaluated quality of life in patients undergoing radiotherapy and demonstrated a significant reduction, manifested by symptoms such as prolonged swelling, speech impairment, and restricted mouth opening [16]. Furthermore, severe complications such as osteoradionecrosis should be considered, which in advanced cases may necessitate extensive surgical interventions, including reconstruction with osseous microvascular free flaps [28, 29].

These potential adverse effects should be carefully weighed against the potential benefits of postoperative adjuvant therapy, alongside conventional survival outcomes.

The findings of our study are based on an exclusively OSCC cohort treated in a comparable manner.

Nevertheless, several limitations must be acknowledged when interpreting the results. First, this is a retrospective study and is therefore subject to the inherent limitations of this design. The limited sample size, particularly after propensity score matching, may have reduced statistical power and increased the risk of overfitting in multivariable Cox regression analyses, which should be taken into account when interpreting the results. Due to the retrospective design and the study period extending back to 2009, relevant adverse pathological features were not consistently available, potentially resulting in residual confounding and selection bias in treatment allocation, which may have influenced the observed hazard ratios. Furthermore, the wide confidence interval for adjuvant therapy in the multivariable analysis reflects the limited sample size and the resulting uncertainty of the effect estimate, and should therefore be considered when assessing the robustness of this finding.

In summary, our data demonstrate an association between adjuvant therapy and improved RFS in patients with pT1–2 pN1 OSCC. In conjunction with the existing literature, these findings support a potential benefit of adjuvant treatment in the pN1 setting. However, given the limited sample size and the associated uncertainty of the effect estimates, these findings should be interpreted with caution. Treatment decisions should therefore be made within a shared decision-making framework, taking into account the individual patient characteristics and the potential adverse effects of radiotherapy or radiochemotherapy.

## Data Availability

All data generated or analyzed during this study are included in this published article.

## Abbreviations

ECS: extracapsular spread
OPSCC: oropharyngeal squamous cell carcinoma
OSCC: oral squamous cell carcinoma
OS: overall survival
PORT: received postoperative radiotherapy
PSM: propensity score matching
RFS: recurrence free survival
